# Adverse events with fatal outcome associated with alemtuzumab treatment in multiple sclerosis

**DOI:** 10.1186/s13104-019-4507-6

**Published:** 2019-08-12

**Authors:** Trygve Holmøy, Børre Fevang, David Benee Olsen, Olav Spigset, Lars Bø

**Affiliations:** 1 Department of Neurology, Akershus University Hospital, Post Office Box 1000, 1478 Lørenskog, Norway; 2 Institute of Clinical Medicine, University of Oslo, Oslo, Norway; 3 Centre for Rare Disorders, Oslo University Hospital, Sognsvannsveien 20, 0372 Oslo, Norway; 4 Section of Clinical Immunology and Infectious Diseases, Oslo University Hospital, Sognsvannsveien 20, 0372 Oslo, Norway; 5 Department of Pharmacovigilance, Norwegian Medicines Agency, Post Office Box 240, Skøyen, 0213 Oslo, Norway; 6 Department of Clinical Pharmacology, St. Olavs University Hospital, Post Office Box 3250, Torgarden, 7006 Trondheim, Norway; 7 Department of Clinical and Molecular Medicine, Norwegian University of Science and Technology, Trondheim, Norway; 8 Norwegian Multiple Sclerosis Competence Centre, Department of Neurology, Haukeland University Hospital, Post Office Box 1400, 5021 Bergen, Norway; 9 Department of Clinical Medicine, University of Bergen, Bergen, Norway

**Keywords:** Multiple sclerosis, Treatment, Alemtuzumab, Adverse event

## Abstract

**Objective:**

Sporadic fatal adverse events have been reported during treatment of multiple sclerosis with alemtuzumab. To provide a systematic overview, we searched the centralized European Medicines Agency database of suspected adverse reactions related to medicinal products (EudraVigilance) for fatal adverse events associated with treatment with alemtuzumab (Lemtrada®) for multiple sclerosis. Four independent reviewers with expertise on MS, clinical immunology, infectious diseases and clinical pharmacology reviewed the reports, and scored the likelihood for causality.

**Results:**

We identified nine cases with a probable and one case with a possible causal relationship between alemtuzumab treatment and a fatal adverse event. Six of these patients died within one month after treatment; one from intracerebral hemorrhage, two from acute multiple organ failure and septic shock, one from listeriosis, one from pneumonia and one from agranulocytosis. Four patients died several months after administration of alemtuzumab from either autoimmune hepatitis, immune-mediated thrombocytopenia, autoimmune hemolytic anemia or agranulocytosis. Four of the 10 cases had been published previously in case reports or congress abstracts. Fatal adverse events related to treatment with alemtuzumab occur more frequently than previously published in the literature. A significant proportion occurs in the first month after treatment.

## Introduction

Alemtuzumab is a humanized monoclonal antibody directed against CD52, and is regarded as one of the most efficacious drugs for treatment of relapsing–remitting multiple sclerosis (MS) [1]. Alemtuzumab induces a profound decrease of T and B lymphocytes, with a gradual recovery starting one month after administration [2, 3]*.* Even though alemtuzumab is generally considered safe, serious adverse reactions have been identified, including infections, immune-mediated thrombocytopenia and thyroiditis [3]. Following regulatory approval of alemtuzumab for relapsing remitting MS in 2013 by the European Medicines Agency (EMA) and in 2014 by the U.S. Food and Drug Administration (FDA), there have been reports of severe and even fatal suspected adverse effects. These include listeriosis [4, 5], alveolar hemorrhage [6], neutropenia with staphylococcus infection [7], autoimmune hemolytic anemia with necrotizing leukoencephalopathy [8], and hemophagocytic lymphohistiocytosis [9].

These concerns led us to perform a systematic search for information on fatal cases following treatment with alemtuzumab in MS, retrieving data from the European database of suspected adverse drug reaction reports (EudraVigilance).

## Main text

### Methods

On November 19, 2018 we searched EudraVigilance for reports with product name “Lemtrada” as the suspect (or interacting) drug, and with the Medical Dictionary for Regulatory Activities (MedDRA) indication high-level term “Multiple sclerosis, acute and progressive”, using the EudraVigilance Data Analysis System (EVDAS). Only reactions classified as “fatal” were included, as well as cases with the reaction outcome “fatal” and with Reaction Seriousness Death set to “Yes”. The search included post-marketing spontaneous reports and reports from clinical studies from the European Economic Area, i.e. the European Union, Iceland, Liechtenstein and Norway.

Four reviewers with clinical and research experience in MS and neuroimmunology (LB and TH), clinical immunology and infectious diseases (BF) and clinical pharmacology (OS), independently reviewed the full Council for International Organization of Medical Sciences (CIOMS) reports and case narratives reports and scored the likelihood for causality in one of the four groups > 85%, 85–50%, 50–35% or < 15%. The cases were then discussed and the fatal adverse event assessed as related or unrelated to alemtuzumab, using guidance from the FDA and the World Health Organization and Uppsala Monitoring Centre [10, 11]. Based on the known safety profile and biological effects of alemtuzumab, we considered immunosuppression, infection or hyperinflammation in close proximity of treatment, as well as secondary autoimmunity occurring months after treatment, as plausible consequences of alemtuzumab. As previously described for the assessment of the association between acute acalculous cholecystitis and alemtuzumab [12], the related cases were further subdivided as either probable or possible from the plausibility and robustness of the evidence, including whether alternative explanations could be reasonably ruled out from the available data. Case reports lacking information essential for the assessment of causality, including the temporal relationship between alemtuzumab treatment and the adverse event, disease history or concomitant medication, or where duplication could not be excluded, were discarded.

### Results

After exclusion of duplicates, including two cases of fatal autoimmune hepatitis occurring almost simultaneously in the same country which were not marked as duplications in the case reports, there were 17 unique cases. In 10 of these (nine female and one male) the fatal adverse events were considered to be related to alemtuzumab. Clinical characteristics of these cases are shown in Table 1. All these patients were adults. The age was not further specified for one case, the others ranged from 34 to 47 years.

**Table 1:** Overview and causality scores of the 10 cases of fatal adverse events considered to have a probable or possible relationship with alemtuzumab, identified in the European Medicines Agency database

Patient no	Gender	Cycle no	Adverse event	Time to symptoms	Death	Causality scores^a^				Conclusion
1	F	1	Intracerebral hemorrhage	5 days	5 days	1	1	1	2	Probable
2	F	1	*Listeria* encephalitis	10 days	12 days	1	1	1	1	Probable
3	F	1	Septic shock, multiple organ failure	3 days	15 days	2	1	2	2	Probable
4	F	1	Septic shock, multiple organ failure	14 days	16 days	1	1	2	2	Probable
5	F	1	Pneumonia	16 days	22 days	2	1	2	2	Probable
6	F	1	Neutropenia, *Staphylococcus aureus* infection, septic shock	27 days	28 days	1	1	1	2	Probable
7	F	1	Autoimmune hemolytic anemia, septic shock, DIC	8 months	8 months	1	1	1	1	Probable
8	M	2	Immune-mediated thrombocytopenia, brain stem hemorrhage	5 months	9 months	2	1	1	1	Probable
9	F	NR	Autoimmune hepatitis	15 months	16 months	2	2	1	2	Probable
10	F	2	Agranulocytosis, *Clostridium* *colitis*, *Aspergillus* *pneumonia*	17 months	18 months	2	1	3	3	Possible

Cases are sorted after time from treatment to death

*DIC* disseminated intravascular coagulation, *VZV* varicella zoster virus, *NR* not reported

^a^Causality scores given by the four individual reviewers, using the following scale: 1: > 85% likelihood for causal relationship with alemtuzumab; 2: 85–50% likelihood; 3: 50–15% likelihood; 4: < 15% likelihood

In nine unique cases (No. 1–9 in Table 1) all reviewers considered that the fatal adverse event was probably to be caused by alemtuzumab. Six of the patients (No. 1–6) died within one month after alemtuzumab infusion. All patients who died within one month had only received one alemtuzumab cycle. Five of these patients (No. 2–6) died from infection or multiple organ failure and septic shock, whereas the sixth (No. 1) developed hypertension and a cytokine storm and died from an intracerebral hemorrhage five days after receiving the first alemtuzumab infusion. Autopsy revealed necrotizing vasculopathy, but did not confirm that the patient had MS. The reporting physician concluded that causality with alemtuzumab was unlikely, as cerebral hemorrhage was not a known adverse event of alemtuzumab. The patient had also received the antithrombotic drug certoparin sodium. Given the immediate temporal relationship and the recent report of early strokes associated with alemtuzumab treatment from the FDA [13], we concluded that causality was probable. Notably, increasing blood pressure, which was reported in this patient, was recently suggested to be a characteristic feature of alemtuzumab-induced stroke [14].

The remaining three patients in whom a causal role of alemtuzumab were considered probable (No. 7–9) were all female, and died from secondary autoimmunity 8 to 18 months after the last alemtuzumab infusion. Of these, one patient died from immune-mediated thrombocytopenia and brain stem hemorrhage, one from autoimmune hepatitis, and one from autoimmune hemolytic anemia, disseminated intravascular coagulation and septic shock. Immune-mediated thrombocytopenia was diagnosed several weeks prior to the intracranial hemorrhage, but did not respond to treatment with corticosteroids and intravenous immunoglobulins.

In five cases the fatal adverse event [suicide (n = 2), cancer (n = 2) and status epilepticus (n = 1)] were considered unlikely to be related to alemtuzumab. In addition, two cases were considered unclassifiable. These were neonates weighing less than 500 g, who both died within one day after induced labor. The mother had been treated with alemtuzumab during pregnancy.

The reviewers disagreed substantially on two cases. One patient (No. 10) developed agranulocytosis 17 months after the second course of alemtuzumab, followed by colitis, aspergillus pneumonia and death from multiple organ failure. CD4^+^ T cells were also low prior to death. Two reviewers scored the likelihood of a causal relationship as < 50%. It was, however, agreed to emphasize that neutropenia grade III or IV have been reported in 1.5% of MS patients in the second year after alemtuzumab treatment [15], and that it was plausible that neutropenia in combination with low CD4^+^ T cell counts induced by alemtuzumab contributed to aspergillus pneumonia and death. A causal relationship with alemtuzumab was therefore considered possible. The second patient, who had aggressive MS, developed status epilepticus few days after alemtuzumab, followed by aspiration pneumonia, sepsis and colon bleeding, and died after 40 days. In spite of a close temporal relationship with alemtuzumab most reviewers considered that status epilepticus was more likely caused by aggressive MS, and that causality therefore was unlikely.

### Discussion

We identified 9 case reports of MS patients with fatal adverse events considered to be probably caused by alemtuzumab, and one with a possible causal relationship. Four of these cases (No. 2 and 6–8) have previously been published or reported on meetings [4, 7, 16, 17], whereas six have not been published. Our results were shared with the Pharmacovigilance Risk Assessment Committee in EMA before EMA initiated an article 20 review of Lemtrada on April 12 2019.

As expected from the pharmacodynamic properties and known adverse drug reaction profile of alemtuzumab, fatal adverse events either occurred early and were characterized by immunosuppression, hyperinflammation or stroke, or occurred several months later and were characterized by secondary autoimmunity. Secondary autoimmunity is known to occur frequently after alemtuzumab treatment in MS patients, and is suggested to be mediated by the reappearance of naïve immunologically active B cells while regulatory T cells are still suppressed [18]. All cases of fatal secondary autoimmunity occurred within a time frame that is compatible with such a hypothesis. It is also known that listeriosis occurs during the first weeks after treatment with alemtuzumab, possibly reflecting marked and transient acute effects on both the adaptive and innate immunity, including impaired function of remaining immune cells [19]. The underlying mechanisms of such acute reactions is believed to include a programmed release of cytokines from natural killer cells, triggered by Fc cross-linking [20].

After we performed our search, FDA issued a warning related to 13 cases of and hemorrhagic stroke or arterial dissection occurring shortly after the patients had received alemtuzumab, mostly within one day after infusion [13]. This, along with the recently published report of eight cases of acalculous cholecystitis in close time proximity to alemtuzumab treatment [12], support a hypothesis of hyperinflammation after alemtuzumab treatment despite routine prophylaxis with corticosteroids.

Immune-mediated thrombocytopenia occurs in approximately 2% of MS patients treated with alemtuzumab [21]. It usually responds well to standard treatment with corticosteroids, although the fatal case reported here indicates that this is not always the case. The finding of only one case of immune-mediated thrombocytopenia with fatal outcome may however suggest that obligate screening for thrombocytopenia clearly limits the consequences of this adverse event. The same may be the case for glomerulonephritis, which was not recorded in this dataset.

Underreporting of adverse events is frequent, and even for severe adverse events it is estimated that only 1–10% of adverse events are reported [22]. Although underreporting may be less frequent for fatal adverse events, a PubMed search identified two fatal cases in Europe that were not registered in EudraVigilance [8, 9]. As some cases are likely neither published nor reported, the number of fatal adverse events in Europe may exceed the 12 events identified by us.

Whereas patients are routinely screened for secondary autoimmunity every month for at least four years after receiving alemtuzumab, the first weeks after treatment have received relatively little attention. Our results indicate that life threatening adverse events could be more frequent during this period. It should, however, be taken into consideration that in general, associations between drug treatment and adverse events are more easily recognized, and therefore also more often reported, shortly after commencement of drug therapy. In our material, the initial events mostly included infections and hyperinflammation. Prophylactic treatment with antibiotics has been suggested in addition to the antiviral therapy that is generally used [23], and could perhaps prevent listeriosis more effectively than diet advice alone. Weekly hematological screening the first period following treatment could possibly limit the consequences of early agranulocytosis [7], and monitoring of blood pressure could possibly prevent early cerebral hemorrhages [14]. Early adverse effects due to hyperinflammation can, however, at present neither be predicted nor fully prevented.

### Conclusions

Fatal adverse events related to treatment with alemtuzumab occur more frequently than previously published in the literature, and seem to be most common during the first month after treatment. Patients, physicians and regulatory authorities should be aware of the serious risks associated with alemtuzumab treatment, which must be weighed against the high and durable clinical efficacy.

## Limitations

- We had not access to full medical records. Although the CIOMS reports were generally quite detailed, we may have missed relevant information.
- The number of multiple sclerosis patients treated with alemtuzumab is not in the public domain. We can therefore not calculate the frequency of fatal adverse events.
- We may have missed cases that have not been reported to EudraVigilance.

## Data Availability

The authors are not permitted to share the case reports. Requests for access to these must be addressed to EMA.

## Abbreviations

CD: cluster of differentiation
CIOMS: Council for International Organizations in Medical Sciences
EVDAS: EudraVigilance Data Analysis System
EMA: European Medicines Agency
FDA: Federal Drug Agency
MS: multiple sclerosis
